# Association of miR-196a-2 and miR-499 variants with ulcerative colitis and their correlation with expression of respective miRNAs

**DOI:** 10.1371/journal.pone.0173447

**Published:** 2017-03-16

**Authors:** Raju Ranjha, Naresh Kumar Meena, Abhiraman Singh, Vineet Ahuja, Jaishree Paul

**Affiliations:** 1 School of Life Sciences, Jawaharlal Nehru University, New Delhi, India; 2 Department of Gastroenterology, All India Institute of Medical Sciences, New Delhi, India; "INSERM", FRANCE

## Abstract

**Background and aim:**

MicroRNAs are small non-coding RNAs that play an important role in regulating the gene expression of their target genes. SNP miR-196a-2 rs11614913 and miR-499 rs3746444 are reported to have association with the risk and prognosis of multiple-types of inflammatory diseases including IBD. This study was conducted to show if any association of SNP miR-196a-2rs11614913 and miR-499 rs3746444 exists with ulcerative colitis (UC) patients of north Indian population and how these polymorphisms modulate the expression profile of the respective miRNAs.

**Methods:**

A total of 638 participants including 197 UC patients and 441 controls were included in this study. Polymorphisms were genotyped by PCR-RFLP and the miRNA expression was measured using qRT-PCR. Genotypes and allele frequencies were calculated using SPSS 16 software.

**Results:**

MiR-196a-2 rs11614913 (C>T) and miR-499 rs3746444 (T>C) were found to be associated with UC. TT genotype of miR-196a-2 rs11614913 (p = 0.03) was negatively associated with UC whereas the heterozygous TC genotype of miR-499 rs3746444 (p = 0.003) was showing positive association with UC. Patients having a combination of both SNPs, developed disease at older age and they suffered from severe disease extent. Genotype that showed association with the disease also showed correlation with the changes in miRNA expression.

**Conclusion:**

In this study we found miR-196a-2 rs11614913 and miR-499 rs3746444 were associated with UC in north Indian population. We found the genotype that showed association with UC also altered the expression of respective miRNA in the patient harboring the genotype. There was correlation between associated genotype and altered miRNA expression.

## Introduction

Inflammatory Bowel Disease (IBD) is a chronic, relapsing and idiopathic inflammatory disorder of gastrointestinal tract. Complex interactions between host immune system, intestinal flora and host genotype are thought to be responsible for the development of IBD [1]. Ulcerative colitis and Crohn`s disease are the two main pathological types of inflammatory bowel disease [2]. UC is characterized by inflammation of the mucosa and occasionally the sub-mucosa and colon part of gastrointestinal tract [3].

MiRNAs are an abundant class of non-coding RNA molecules that are emerging as key players in regulating gene expression [4]. MiRNAs regulate the expression of 10–30% of all human genes and have been shown to play critical roles in the coordination of cell differentiation, proliferation, death, metabolism and tumorigenesis [5]. MiRNA dysregulation has been reported in many diseases including inflammatory diseases and various cancers [6].

Recently genetic variations, mainly single nucleotide polymorphism (SNP) has been reported in pre- or mature miRNA sequences that are found to affect the processing and function of miRNA [7–8]. In a population, miR-polymorphisms can be present either in a heterozygous or homozygous configuration, in the form of insertions, deletions, amplifications or chromosomal translocations, resulting in loss or gain of a miRNA site/function.

Single Nucleotide polymorphism (SNP), the most important genetic marker, located in miRNA genes may affect the processing and functioning of miRNA that may contribute to the susceptibility towards disease development. A large number of studies show SNPs miR-196a-2 rs11614913 and miR-499 rs3746444 present in miRNA genes to be associated with various diseases including Rheumatoid arthritis, colorectal cancer, renal cell cancer and schizophrenia as reported from China and other countries of Asia. [9–15]. A meta-analysis suggested that hsa-mir-499 rs3746444 T > C polymorphism is associated with the risk of cancer in Asians, mainly in Iranian and Chinese population. However, rs3746444 T > C polymorphism was negatively associated with the risk of esophageal cancer [16]. In Japanese population heterozygous miR-499 rs3746444 was found to be associated with UC and miR-196a-2 rs1161913 TT genotype held a significantly higher risk of refractory phenotype [17]. A protective role of TT genotype of miR-196a-2 rs1161913 was observed against UC in Greek population [18]. SNP in miRNA may affect its own expression and subsequently the expression of its target gene [15, 19]. Any study on association of these SNPs with UC and the affect of SNPs on miRNA expression so far has not been reported from Indian population.

MiRNA sequence alteration can affect miRNA processing as well as its binding to target mRNA. MiR-499 rs3746444 SNP is present in the seed region of mature miRNA sequence and miR-196a-2 rs1161913 is present in the non-seed region of mature miRNA. So these SNPS can affect the target binding of the respective miRNAs. Functional alteration in miRNA may affect the pathogenesis of UC. We carried out this case control study to score these SNPs in our population by PCR-RFLP studies. Further, relative expression of hsa-miR-196a-2 and hsa-miR-499 was analyzed with respect to their genotypes in patients and control by qRT-PCR.

## Materials and methods

### Study population

The study population included 197 ulcerative colitis patients and 441 control participants. The study was approved by ethical committee of All India Institute of Medical Sciences, New Delhi, India (Ref. No.—IEC/NP-320/2012) and Institutional Ethics Review Board, Jawaharlal Nehru University, New Delhi (REF no. 2012/student/28). The diagnosis of UC were established according to clinical guidelines based on Montreal classification [20]. The total index score was used to calculate the specific index and severity of disease [21]. Age and sex matched control subjects with no history of IBD or any other inflammatory disease were included in the study. Demographic features of UC patients and controls are described in Table 1. 2 ml of venous blood sample was collected from each subject in vacutainer tubes (BD, NJ, USA) containing the anticoagulant K_2_EDTA solution. Pinch colon biopsy samples were obtained from the UC patients (n = 34) and non-IBD controls (n = 25) undergoing colonoscopy in 1.5 ml tubes containing RNA later solution (Sigma-Aldrich, MO, USA). Colon biopsy samples were collected from sigmoid and left colon of the patients. Biopsy samples from active UC patients with total index score >07 were used in the present study. Written consent was taken from all the subjects who participated in this study.

**Table 1:** Demographic features stratified in UC cases and control group.

Demographic features	UC (n = 197)	Control (n = 441)
**Age Mean**± SD (Range)	35.51± 13.20 (19–68)	34.14±12.74 (20–65)
**Sex (M/F)**	117/80	267/174
**Duration of disease, mean**± SD **(range in years)**	5.38± 5.42 (0.1–30)	NA
**Age at diagnosis (yr)**		
15–40	130 (65.99%)	NA
>40	67 (34.01%)	NA
**Disease extent, n (%)**		
Rectum	23 (11.67%)	NA
Proctosigmoditis	49 (24.87%)	NA
Left sided	47 (23.85%)	NA
Pancolitis	78 (39.59%)	NA
**Social Status**		
Rural	101 (51.27%)	219 (49.66%)
Urban	96 (48.73%)	222 (50.34%)
**Smoking history**		
Yes	18 (9.13%)	71 (16.1%)
No	179 (90.86%)	370 (83.9%)
**Appendectomy Y/N (%)**	4/197 (2.03%)	0/441

SD- Standard Deviation, M-Male F- Female Yr-Year n-number Y-yes N-no

### DNA isolation and genotyping

**Fig 1:**
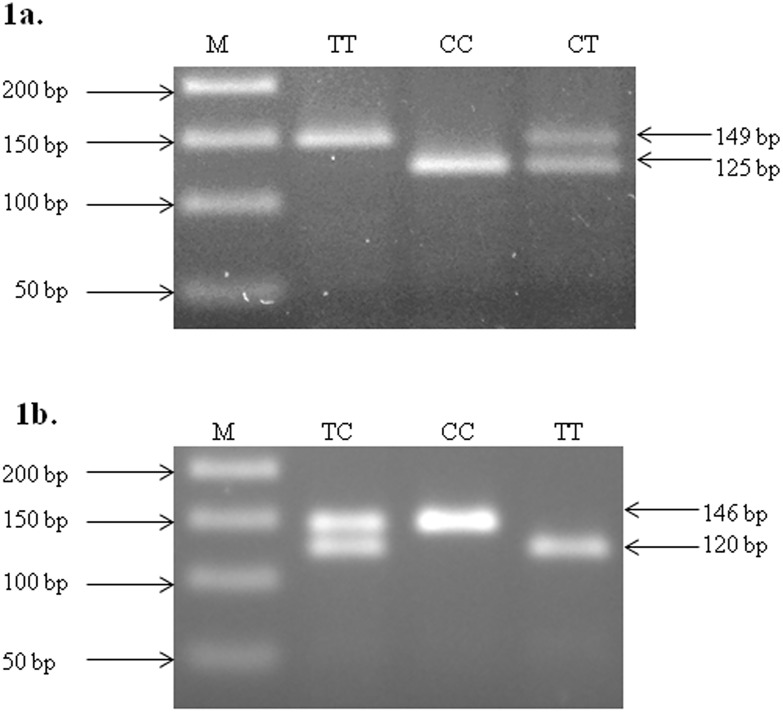
RFLP profiling of hsa-miR-196a-2 rs11614913 C>T (a) and hsa-miR-499 rs 3746444 T>C (b). **1a**- 146 bp DNA fragment was amplified using PCR and incubated with MspI at 37°C for 12–16 hrs. Hsa-miR-196a-2 rs11614913 C>T genotype were deduced from migration profile on 2% agarose gel electrophoresis. Lane M shows 50bp molecular marker, wild type DNA visible in lane CC, heterozygous mutant in lane CT and homozygous mutant in lane TT. **1b**- 149 bp DNA fragment was amplified using PCR, incubated with BclI at 37°C for 12–16 hrs, hsa-miR-499 rs 3746444 T>C genotype were deduced from migration profile on 2% agarose gel electrophoresis. Lane M shows 50bp molecular marker, wild type DNA visible in lane TT, heterozygous mutant in lane TC and homozygous mutant in lane CC.

Genomic DNA was isolated from peripheral blood leucocytes using phenol chloroform method described by Miller et al. [22]. Genotyping was performed by polymerase chain reaction-restriction fragment length polymorphism (PCR-RFLP) method. The primers used were as described by Hu et al. (2008), validated by sequencing (data not shown). PCR was performed in a Touch gene PCR machine (Nugen Scientific, USA). Thin walled 0.2ml tubes were used for amplification. Briefly, 20 μL PCR mixture contained 1 μl of genomic DNA (50–70 ng/μl), 1 μl of both forward and reverse primers (20pmol), 2 μl 10x PCR buffer (containing 750mM Tris-HCl (pH 8.8 at 25°C), 200mM (NH4)_2_ SO4, 0.1% Tween-20), 2 μl dNTPs (containing 2mM of each dNTP) and 0.2 μl Taq DNA polymerase (5U/ μl) (MBI Fermentas, USA). The amplification conditions were: one cycle of initial denaturation at 94°C for 5 min. 30 cycles of 94°C for 30s, annealing 63°for 30s, 72°C for 30s and one cycle of final extension at 72°C for 7 min. and finally cooled down to 4°C. The reaction containing all reagents except the template DNA was treated as a negative control. The size and integrity of the products were checked by electrophoresis of 10 μl of the sample on a 1.5% agarose gel at 5 V/cm for an appropriate time period. The PCR product size was 149 bp and 146 bp for miR-196a-2 rs11614913 and miR-499 rs3746444 respectively. Restriction digestion was carried out with 10U of MspI and BclI (Thermo Fisher Scientific, USA) restriction enzymes respectively at 37°C for 12–16 hr as per manufacturer’s instructions. Following incubation, the digested product was run on 2% agarose gel containing ethidium bromide (EtBr). Representative gel picture showing the restriction digested PCR products for two SNPs observed in the miRNAs are shown in Fig 1. A small fragment of 24bp for miR-196a-2 rs11614913 and 26bp in case of miR-499 rs3746444 genotyping was not visible on agarose gel electrophoresis.

### RNA isolation and quantitative Real Time PCR (qRT-PCR)

Total RNA was isolated from the biopsy samples using mirVana miRNA isolation kit (Ambion INC, TX 78744, USA) and stored at -80°C. RNA was quantified by NanoDrop ND-1000 spectrophotometer (Thermo scientific, USA). RNA quality was assessed by running 1.5% agarose gel. RNA was reverse transcribed using gene specific looped primers [23] by revert aid cDNA synthesis kit (Thermo Fisher Scientific, Waltham, Massachusetts, USA). Real time PCR was performed using ABI PRISM 7500 Real time PCR system (Applied Biosystems). The parameters followed were: Initial denaturation—94°c for 2 min, denaturation- 94°c for 30 sec, annealing- 60°c for 1 min for 40 cycles. 20 μl reaction containing 7 μl MQ, 10 μl SYBR^R^ Green universal PCR Master Mix (Applied Biosystems, California, USA), 1 μl of each forward and reverse primer (4 picomole/ μl), 1 μl cDNA. Sequences of primers used are given in Table 2. Relative expression of miRNA was calculated using Ct values. U6 snRNA was used as an internal control for miRNA expression analysis. GAPDH was used as internal control for expression analysis of other genes.

**Table 2:** Sequence of primers used for reverse transcription and real time PCR.

Primer name	Specification	Primer sequence
Hsa-miR-196a-2	RT primer	GTCGTATCCAGTGCAGGGTCCGAGGTATTCGCACTGGATACGCTCAGGCA
	Forward primer	CGGCAACAAGAAACTGCCTG
Hsa-miR-499	RT primer	GTCGTATCCAGTGCAGGGTCCGAGGTATTCGCACTGGATACGAGCACAGA
		GAACATCACAGCAAGTCTGA
U6 SnRNA	RT primer	GTCGTATCCAGTGCAGGGTCCGAGGTATTCGCACTGGATACGAAAATATG
	Forward primer	CAAATTCGTGAAGCGTTCCA
Myosin heavy Chain 7B (MYH7B)	Forward	GTCTGGGTGCCTGATGAACA
	Reverse	CTCGTTCAGGTGCGTCATCA
HOXC8	Forward	CGCACCACGTTCAAGACTTCT
	Reverse	TAAGCGAGCACGGGTTCTG
HOXC-AS1	Forward	CTGCGACACTTCCCCACC
	Reverse	CTACTTGCCCACGACCGA

### Statistical analysis

SPSS version 16.0 software (SPSS Inc., Chicago, Illinois, USA) and Graph Pad Prism version 5 (San Diego, California: Graph Pad Software Inc., 2007) were used to perform statistical analysis. Two-tailed p values ≤ 0.05 were considered significant. Hardy-Weinberg equilibrium was carried out to determine whether the proportion of each genotype obtained was in agreement with expected values as calculated from allele frequencies. Detailed analysis of the observed genotypes in both controls and patient samples was performed using various tests such as, Pearson Chi Square, Fisher’s exact test, Student’s t test and ANOVA.

Minor allele frequency was calculated using following formula:

MAF= (2× M) + H/T

M = Number of mutant individuals in population considered

H = Number of heterozygous individuals in the given population

T = Total number of alleles in the given population

The relative expression was calculated using the equation 2^-ΔΔCT^ where ΔΔCT = Δ CT target−ΔCT internal control. All statistical tests were two-sided, and a probability level of p ≤ 0.05 was considered statistically significant.

## Results

### Characteristics of study subjects

Study subjects were unrelated north Indian population and were recruited to the Department of Gastroenterology, All India Institute of Medical Sciences, New Delhi India. Demographic details showed that 65.99% of the cases developed UC at early age, (younger age group< 40 years). Diseases showed a negative association with smoking status of patients as 90.86% of the UC patients was non smokers. Only 2.03% patients had appendectomy. The percentage of patients having severe form of disease was higher (pancolitis, 39.59%) compared to less sever disease (proctitis, proctosigmoditis, left sided colitis).

### Association of miR196a-2 and miR-499 polymorphisms with ulcerative colitis

The frequency distribution of miR196-a2 rs11614913 and miR-499 rs3746444 genotype and risk of UC are given in Table 3. TT genotype of miR-196a-2 rs11614913(C>T) showed a negative association with UC compared to CC genotype, p = 0.031, OR = 0.497 (CI = 0.264–0.935). MiR-499 rs3746444 heterozygous TC genotype was found to be significantly associated with increased risk of UC, p = 0.000, OR = 3.651 (CI = 2.362–5.645). The allele frequencies were in Hardy-Weinberg equilibrium both in patients and control groups.

**Table 3:** Association between miR196a-2 rs11614913 and miR-499 rs3746444 SNP with the susceptibility to UC.

Genotype	Control	Patient	OR (95% CI)	P value
**rs11614913 (C>T)**				
CC	305 (69.16%)	102 (51.77%)		
CT	81 (18.36%)	81 (41.11%)	0.886 (0.622–1.262)	0.529
TT	55 (12.47%)	14 (7.1%)	0.497 (0.264–0.935)	0.031
MAF	21.65%	27.66%		
**rs3746444 (T>C)**				
TT	167 (37.86%)	97 (49.23%)		
TC	220 (49.88%)	35 (17.76%)	3.651 (2.362–5.645)	0.000
CC	54 (12.24%)	65 (32.99%)	0.483 (0.311–0.749)	0.001
MAF (%)	37.18%	41.88%		

OR- Odds Ratio, MAF- Minor Allele Frequency, CI- Confidence Interval

### Genotype phenotype correlation analysis for combination of SNPs

In order to draw genotype-phenotype correlations, genotype and allele frequencies of observed SNPs were stratified by phenotypic sub groups of UC patients (Table 4).

**Table 4:** Genotype-phenotype correlation of SNPs with risk factors of UC.

SNP/Geno-type	Sex		Age at Diagnosis (Yrs.)		Disease Extent				Smoking history		Social Status	
	Male	Female	15–40	>40	Proctitis	Procto-sigmoditis	LeftSided Colitis *	Pancolitis	Yes	No	Urban	Rural
CC/TT (W)	110	75	123	62	22	47	41	75	17	168	94	91
CT/TC (M)	6	3	4	5	0	2	5	2	1	8	5	4
Total	116	78	127	67	22	49	46	75	18	176	99	95
MAF (%)	5.17	3.85	3.15	7.46	0	4.08	10.87	2.67	5.56	4.54	5.05	4.21

MAF- Minor allele Frequency, Yr-years, W-wild, H-Heterozygous, M-Mutant

* Left sided colitis showed positive association with UC p = 0.03, OR = 4.39 (1.13–17.01).

#### MiR196a-2 rs11614913 CT/ MiR-499 rs3746444 TC

Patients having combination of SNPs hsa-miR-196a-2 rs11614913 CT/ hsa-miR-499 rs3746444 TC were subjected to genotype phenotype correlation. No association of genotype was observed with sex, age at diagnosis, smoking history, social status of the patients. MAF was high in left sided colitis (10.87%) compared to proctosigmoditis (4.08%), pancolitis (2.67%) and proctitis (0%). Mutant genotype Combination (CT/TC) showed association with left sided colitis p = 0.03, OR = 4.39 (1.13–17.01). We also found three patients with combination of SNPs in homozygous mutant conditions for hsa-miR-196a-2 rs11614913 TT/ hsa-miR-499 rs3746444 CC. However, genotype- phenotype correlations could not be performed for this combination of samples due to low sample size.

### MiR-196a-2 and miR-499 expression changes between control and patients

**Fig 2:**
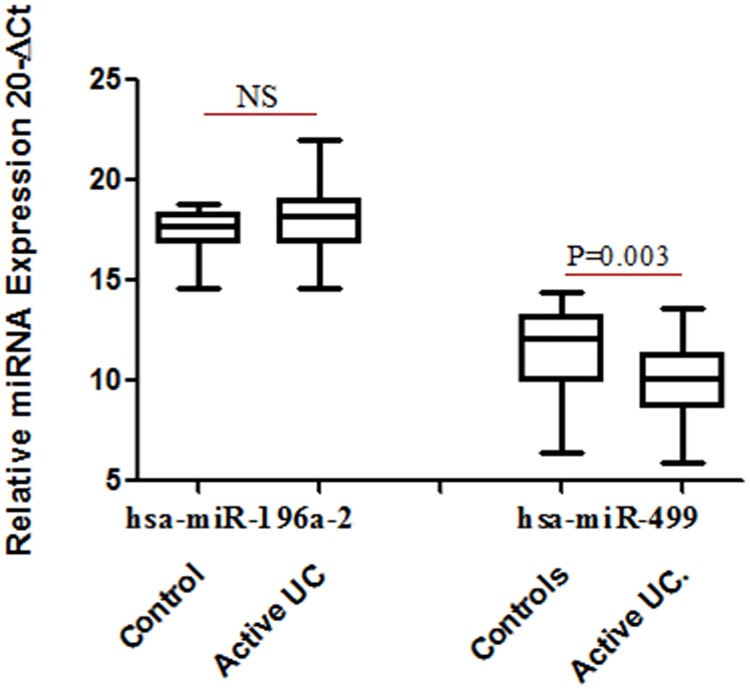
Relative expression of miRNA in UC patients (n = 28) and control subjects (n = 19). Total RNA was isolated from colon biopsy samples from healthy subjects and UC patients and reverse transcribed to cDNA using specific primers. Data derived from quantitative real-time PCR. Normalization of samples was performed with the small nuclear U6 snRNA. Box plots present median ± 25th and 75th percentiles (solid box) with the lowest and highest percentiles shown by whiskers outside the box. The Ct values were subtracted from 20, so that higher values represented higher mRNA expression levels. * represent p ≤ 0.05.

There was no significant change in expression of miR-196a-2 when compared between patient and control group. However expression of miR-499 exhibited a 4 fold decrease (p = 0.003) in UC patients compared to controls indicating the influence of the disease (Fig 2).

### MiR-196a-2 and miR-499 expression changes with allelic variants

**Fig 3:**
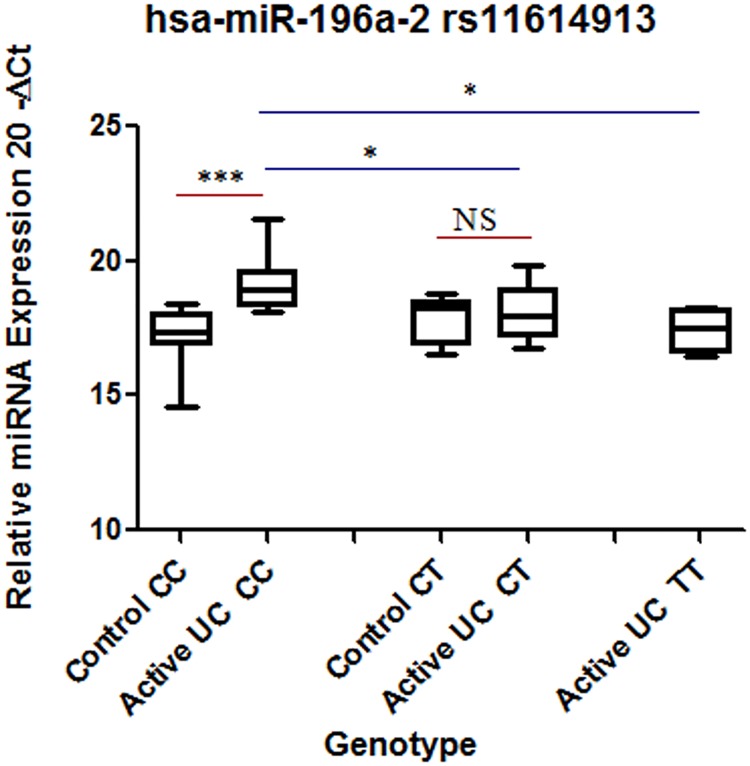
Comparison of hsa-miR196a-2 expression at genotype level between controls and patients. Total RNA was isolated from colon biopsy samples from healthy subjects and UC patients and reverse transcribed to cDNA using specific primers. Data were derived from quantitative real-time PCR. Normalization of samples was performed with the U6 snRNA. Sample size was 8–12 samples in homozygous CC and heterozygous CT in each category. For homozygous mutant n = 4 for UC. Box plots present median ± 25th and 75th percentiles (solid box) with the lowest and highest percentiles shown by whiskers outside the box. The Ct values were subtracted from 20, so higher values represented higher mRNA expression levels. Significance values are represented as * p ≤ 0.05, *** p ≤ 0.001.

Interestingly, when both the patients and controls were segregated at the genotype level, altered expression pattern was observed for both the miRNAs. In individuals with CC genotype (wild type), expression of miR-196a-2 was 3.7 fold higher in patient, compared to controls. However in case of CT genotype, there was no significant change in expression of miR-196a-2 between patients and controls. We had no biopsy samples in control category with TT genotype. Within patients there was a significant decrease in expression of miR-196a-2 in heterozygous CT and mutated homozygous TT genotype compared to homozygous (wild type) CC (Fig 3).

**Fig 4:**
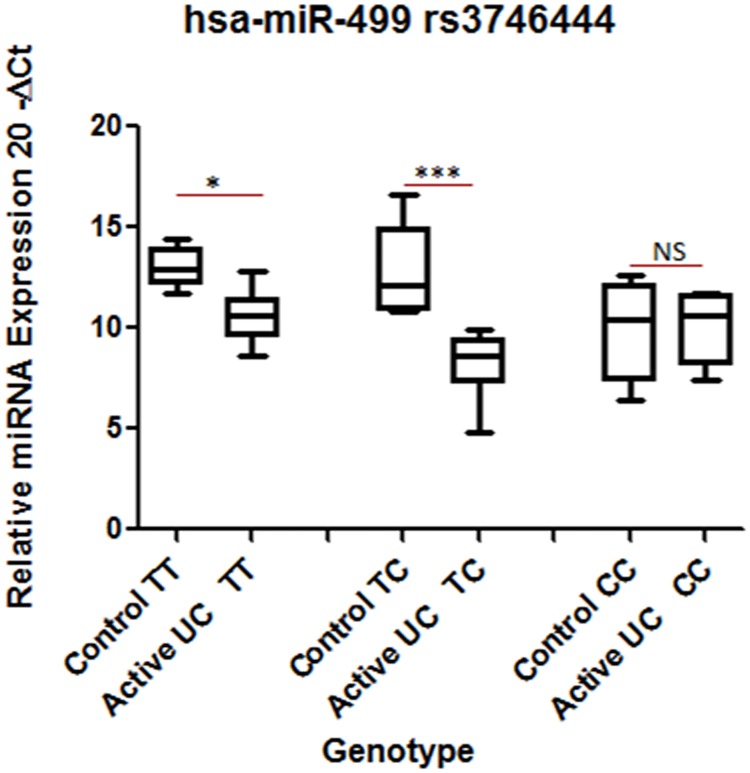
Comparison of hsa-miR-499 expression at genotype level between controls and patients. Total RNA was isolated from colon biopsy samples from healthy subjects and UC patients that were reverse transcribed to cDNA using specific primers. Data were derived from quantitative real-time PCR. Normalization of samples was performed with the small nuclear U6 snRNA. Sample size was 4–12 in each category. Box plots present median ± 25th and 75th percentiles (solid box) with the lowest and highest percentiles shown by whiskers outside the box. The Ct values were subtracted from 20, so that higher values represent higher mRNA expression levels. * represent p ≤ 0.05, *** represent p ≤ 0.001.

The expression of miR-499 decreased significantly by 5.5 fold in patients compared to controls having TT (wild type) genotype. Similarly, in heterozygous condition (TC genotype), the expression decreased by 24.39 fold in patients compared to controls. In Homozygous mutant (CC) condition, there was no significant change in expression of the above miRNA in control vs. UC (Fig 4). MiR-499 expression significantly decreased (5 fold) in patients with TC genotype compared to patients with TT genotype. MiRNA expression did not alter significantly when TT and TC genotypes were compared within patients (Fig 4).

We also looked at the miR-196a-2 and miR-499 expression level at genotype level irrespective of physiology (disease or control) of participant. There was no significant difference in miRNA expression.

#### Effect of SNP on the expression of other surrounding genes

We also looked at the expression level of surrounding genes where these SNPs are located (http://www.gtexportal.org/home/). Expression of HOXC8 and HOXC-AS1 (for rs11614913) and MYH7B (for rs3746444) were analyzed considering the SNPs present. We did not find any significant alteration in the expression of these genes based on genotype (S1a, S1b and S1c Fig).

## Discussion

Recently there has been increasing interests in dissecting out the polymorphism in miRNA genes to understand their crucial role in various disease pathogenesis. In this study we carried out the association study of rs11614913 in miR-196a-2 and rs3746444 in miR-499 with respect to UC patients in north Indian population. We also looked at the effect of these SNP on the expression of corresponding miRNAs. We observed that TT genotype of rs11614913 was negatively and TC genotype of rs3746444 was positively associated with UC in north Indian population. Expression of miR-196a-2 and miR-499 changed significantly due to the allelic variants.

MiR-196a-2 rs11614913 and miR-499 rs3746444 has been discussed in relation to various cancers and inflammatory diseases. Meta-analysis study carried out on 8361 cancer patients and 8504 controls showed association of rs11614913 and rs3746444 with breast cancer, OR = 0.89, 1.13 respectively [24]. A meta analysis conducted by Wang et al on five case control studies, reported negative association of miR-196a-2 rs11614913 and no association of miR-499 rs3746444 with lung cancer [25]. In various Asian and Caucasian populations, C allele of miR-196a-2 rs11614913 was found to be associated with increased susceptibility to CRC, HCC and colorectal cancer [26–27]. TT genotype of miR-196a-2 rs11614913 and CC genotype of miR-499 rs3746444 are reported to be associated with decreased risk of Chronic Obstructive Pulmonary Disease (COPD) [28]. TC genotype of miR-499 rs3746444 has been reported to be associated with higher C-reactive protein (CRP) and erythrocyte sedimentation rate (ESR) compared to CC and TT genotype in rheumatoid arthritis patients [15].

A study carried out in Indian population, described CT genotype of miR-196a-2 rs11614913 as a risk factor for bladder cancer [29]. miR-196a-2 rs11614913 TT genotype is reported to play a protective role in UC in Greek population [18]. Masaaki Okubo et al. have reported that heterozygous miR-499 rs3746444 as a risk factor for UC. They also reported miR-196a-2 rs11614913 may influence the pathophysiological features of UC and is associated with refractory phenotype of UC in Japanese population [17]. In our study, we found heterozygous TC for miR-499 rs3746444 showed a positive association with UC (OR = 3.651, CI = 2.362–5.645, p = 0.000), similar to Greek population. TT genotype miR-196a-2 rs11614913 showed decreased risk of UC compared to CC (OR = 0.497, CI = 0.264–0.935, p = 0.031) (Table 3). Our study supports the earlier reports by Gazouli et al (2013) for miR-196a-2 rs11614913 and Masaaki Okubo et al (2010) for miR-499 rs3746444.

UC is a disease in which different pathogenic mechanisms lead to different clinical subtypes. There are reports that show that different UC subgroups may have different genetic background. There is no data for combined association of these SNPs with UC phenotypes. But rs3746444 AG and rs1161913 TT genotypes were reported to be associated with more severe and rs3746444 AA genotype was reported to be associated with mild UC phenotype. We investigated association of combination of SNPs in two miRNAs with different phenotype of UC. Combination of these SNPs also showed association with severe disease phenotype, left sided colitis OR = 4.39 (1.13–17.01).

MiRNA expression is reported to be tissue specific [30]. It is also observed to be site specific within same tissue due to disease activity [6]. SNPs were implicated in altered expression or biological functions of mature miRNAs [31]. Here we have elucidated how genotype of an individual may be affecting miRNA expression in control and patients differently due to disease activity. The CC homozygous SNP rs11614913 located in miR-196a-2 was associated with a statistically significant increase in the expression of miR-196a-2 in non small cell lung cancer as well as colorectal cancer, [7–8]. Mark Bloomston et al earlier reported increased expression of miR-196a-2 in pancreatic cancer and showed it to be useful marker for prediction in survival of pancreatic cancer patients [32]. In Saudi Arabian population, Alshatwi et al noticed increased expression of miR-196a-2 and miR-499 in breast cancer patients compared to control. They reported decrease in expression from homozygous CC to heterozygous CT and TT for miR-196a-2 and decreased expression from homozygous TT to heterozygous TC and CC for miR-499 [19]. We did not find any significant difference in miR-196a-2 expression between UC and control group when the samples were not categorized on the basis of genotypes. But we found decreased expression of miR-499 in UC compared to controls. Our results indicated that expression pattern of miR-196a-2 and miR-499 was significantly affected by different genotypes. We observed that when UC patients were segregated on the basis of genotype, expression of miR-196a-2 was significantly decreased for heterozygous CT and homozygous TT compared to homozygous CC as reported by Alshatwi et al [19]. No such pattern was observed in the expression of miR-499 in UC patients.

There is no report that shows miRNA expression difference between control and diseased individuals at genotype level. We segregated our samples according to genotype and looked for expression differences. In CC genotype (wild) expression of miR-196a-2 was significantly higher in UC patients compared to controls with the same genotype. Expression level decreased in patients with heterozygous genotype (CT) and further decreased in patients with homozygous mutant TT genotype. We observed that expression level of the miR-196a-2 in mutant TT genotype was comparable to the controls having CC (wild) genotype. In our association analysis (Table 2) we observed TT genotype of miR-196a-2 rs11614913 to be protective OR = 0.497 (CI = 0.264–0.935). We found the genotype that was showing negative association with UC also showed decrease in expression level comparable to non-IBD controls (Fig 3).

In case of miR-499 rs3746444 SNP, results of association and expression analyses showed a correlation in their pattern. In our association analysis, we found heterozygous TC genotype showed positive correlation OR = 3.651 (2.362–5.645) whereas homozygous mutant showed a negative correlation with UC OR = 0.483 (0.311–0.749) (Table 3). In expression analysis the pattern was reflected as we observed ~24.39 fold increase in expression in heterozygous TC genotype between control and patients but was only ~5.5 fold in wild type genotype. Interestingly, expression difference was decreased and there was no significant difference in expression between control and UC patients with mutant CC genotype. Heterozygous TC genotype of miR-499 rs3746444 SNP increased the disease risk in our association analysis OR = 3.651 (2.362–5.645) and also increased the miR-499 expression difference between control and UC patients. However, in homozygous mutant CC of miR-499 rs3746444 SNP exhibited a decreased risk in our association analysis OR = 0.483 (0.311–0.749) and also decrease in expression comparable to non-IBD individuals with same genotype.

Altered behavior of heterozygous and homozygous mutants both in association studies and expression analysis of respective mutants observed by us for the first time in UC patients corroborated the findings of Yang et al, 2012 where they observed differently changed levels of inflammatory markers for heterozygous and homozygous miR-499 rs3746444 SNP in rheumatoid arthritis patients [15]. SNP for miR-499 is present in the seed region of miRNA. Because each mature miRNA variant may target different set of genes so expression of different genes will be affected by mature miRNA of CC and TT homozygotes. For heterozygous CT miR-499 will be having both the SNPs miR-499*C and miR-499*T. So in miR-499 CT will be affecting more target genes in the transcriptome than miR-499 CC and miR-499 TT. Regulation of different target genes by heterozygotes compared with homozygotes may explain the predisposition to the disease as proposed earlier by Jazdzewski K, 2009 in case of polymorphic nature of pre-miR-146a in case of thyroid cancer patients [33]. Contrasting behavior of heterozygous rs3746444 AG and homozygous AA genotype was also reported by Okubo et al, 2011. They found that contrasting association of homozygous and heterozygous rs3746444 with number of times hospitalized, steroid dependence and refractory phenotypes [17]. Contrasting behaviors of heterozygous and homozygous mutant miR-499 rs3746444 SNP reported by Okubo et al and observed in our study. This may be attributed to the location of SNP in miRNA in the seed region and its affect on the target binding and expression.

miR-196a-2 is known to be a part of HOX cluster and location of miR-499 is within the Myosin Heavy Chain 7B (MYH7B) gene. So we also looked at the expression of other eQTL for the two SNPs as shown by GTEX portal (http://www.gtexportal.org/home/). We checked the expression of MYH7B in subjects with SNP rs3746444 and Hox cluster genes, HOXC8 and HOXC-AS1 in subjects having SNP rs11614913. We did not find any significant change in expression of these genes with respect to genotype of the miRNA.

Expression of miR-196a-2 and miR-499 did not show any change when only genotype was considered irrespective of pathological condition of the individual. When expression was compared with respect to genotype both in control vs. patients, significant changes were observed. We propose that the pathology of the individual (diseased or healthy state) along with the genotype contribute in differential expression of miRNA. The changed miRNA expression level may further affect the expression of its target genes.

The present study concludes that TT genotype of miR-196a-2 rs11614913 is negatively and TC genotype miR-499 rs3746444 is positively, associated with UC. Both these SNPs cause alteration in respective miRNA expression according to physiology and genotype of the individual.

### Conclusion

The present study concludes that miR-196a-2 rs11614913 and miR-499 rs3746444 are associated with UC. Both these SNPs cause alteration in respective miRNA expression. Altered miRNA expression showed correlation with genotype that supported association analysis. The influence of the disease state on the expression of mature miRNA was quite intriguing and needs further elaboration. Future studies are warranted to validate these findings in UC and other diseases.

## Supporting information

S1 Fig Comparison of expression of genes present surrounding the SNP rs11614913 and rs3746444. Total RNA was isolated from colon biopsy samples from healthy subjects and UC patients and reverse transcribed to cDNA using random hexamer primers. Data derived from quantitative real-time PCR. Normalization was performed with the GAPDH. Sample size was 4–8 in each category. rs11614913 is present in the HOX cluster. We did not find any significant change in the expression in the expression of HOXC8 (S1a Fig) and HOXC-AS1 (S1b Fig). We had no RNA samples left for mutant genotype so the expression in homozygous wildtype and heterozygous mutant was analysed. SNP rs3746444 is located in the MYH7B gene. We did not find any significant change in expression of MYH7B with respect to genotype (S1c Fig).
(TIF)
