# Correction: Association of miR-196a-2 and miR-499 variants with ulcerative colitis and their correlation with expression of respective miRNAs

**DOI:** 10.1371/journal.pone.0184701

**Published:** 2017-09-07

**Authors:** Raju Ranjha, Naresh Kumar Meena, Abhiraman Singh, Vineet Ahuja, Jaishree Paul

The following information is missing from the Funding section: The authors greatly acknowledge the financial support from Jawaharlal Nehru University, New Delhi, India towards the publication charges of this article.
