# Supplementary figures and images for: Association of miR-196a-2 and miR-499 variants with ulcerative colitis and their correlation with expression of respective miRNAs
Source: PLoS One. 2017 Mar 16;12(3):e0173447. doi: 10.1371/journal.pone.0173447 (PMC5354276; doi:10.1371/journal.pone.0173447)

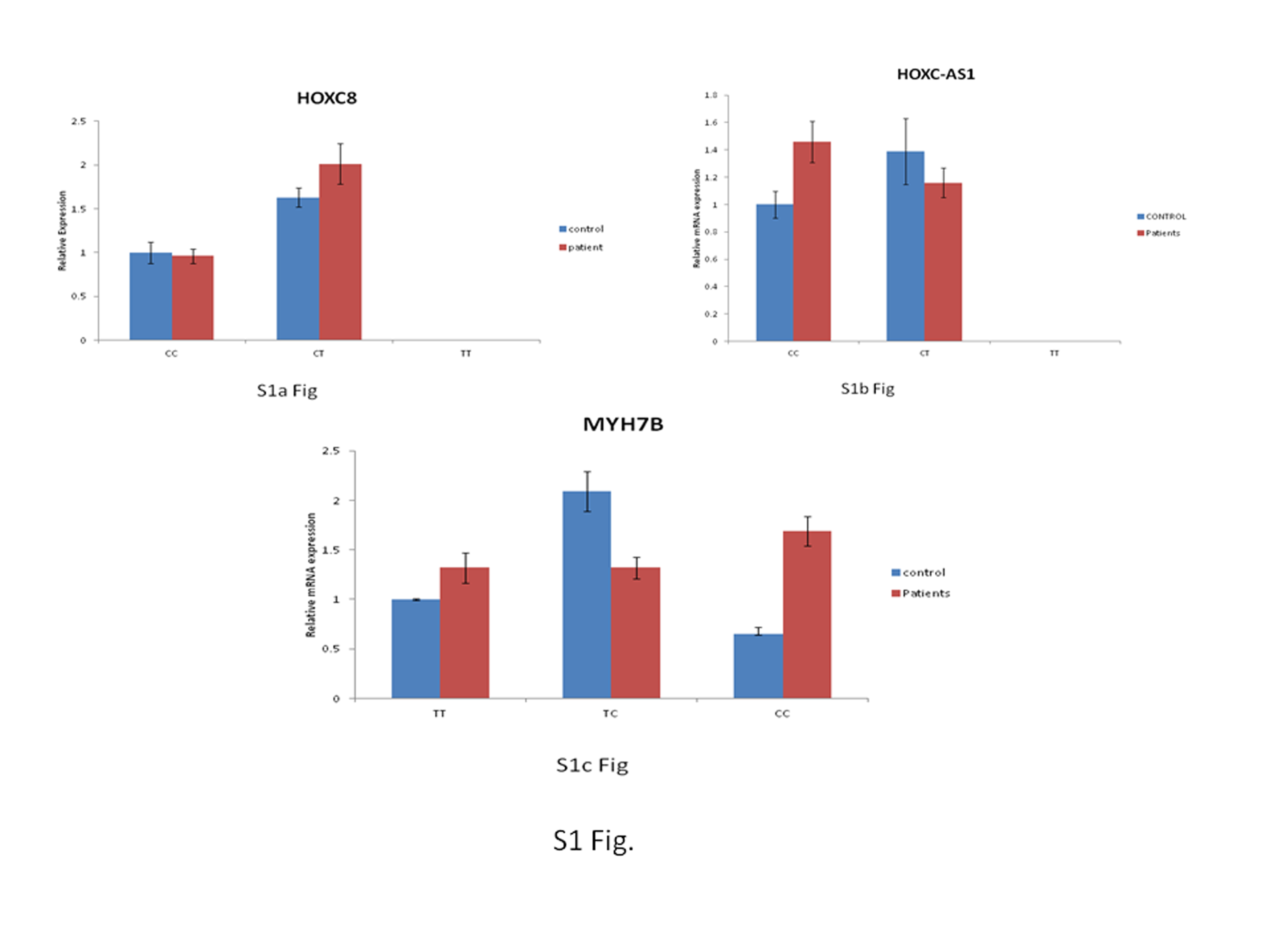

Supplement: S1 Fig — Total RNA was isolated from colon biopsy samples from healthy subjects and UC patients and reverse transcribed to cDNA using random hexamer primers. Data derived from quantitative real-time PCR. Normalization was performed with the GAPDH. Sample size was 4–8 in each category. rs11614913 is present in the HOX cluster. We did not find any significant change in the expression in the expression of HOXC8 (S1a Fig) and HOXC-AS1 (S1b Fig). We had no RNA samples left for mutant genotype so the expression in homozygous wildtype and heterozygous mutant was analysed. SNP rs3746444 is located in the MYH7B gene. We did not find any significant change in expression of MYH7B with respect to genotype (S1c Fig). (TIF) [file pone.0173447.s001.tif]
